# Molecular Monitoring in Adult Philadelphia Chromosome-Positive Acute Lymphoblastic Leukemia with the Variant e13a3 *BCR-ABL1* Fusion

**DOI:** 10.1155/2019/9635070

**Published:** 2019-06-27

**Authors:** Mireille Crampe, Laura Kearney, David O'Brien, C. Larry Bacon, Derville O'Shea, Stephen E. Langabeer

**Affiliations:** ^1^Cancer Molecular Diagnostics, St. James's Hospital, Dublin 8, Ireland; ^2^Department of Haematology, St. James's Hospital, Dublin 8, Ireland; ^3^Department of Haematology, Cork University Hospital, Cork, Ireland

## Abstract

Monitoring *BCR-ABL1* transcript levels in patients with Philadelphia chromosome-positive acute lymphoblastic leukemia (Ph+ ALL) is a widely adopted method to assess response to therapy. However, a small minority of Ph+ ALL patients express variant *BCR-ABL1* transcript types, usually due to splicing of alternative *BCR* or *ABL1* exons. Whether patients expressing these rare, variant *BCR-ABL1* transcripts have a distinct phenotype or response to therapy is not known due to the limited number of reported cases. Here, we report the presenting features of Ph+ ALL in a young adult with a variant e13a3 *BCR-ABL1* fusion. Molecular monitoring reflected the disease response from diagnosis through allogeneic stem cell transplantation which resulted in undetectable e13a3 *BCR-ABL1* transcripts. This case highlights the value of molecular monitoring in Ph+ ALL patients with variant *BCR-ABL1* transcripts and the requirement for standardization of such assays.

## 1. Introduction

Philadelphia chromosome-positive acute lymphoblastic leukemia (Ph+ ALL) in adults is an aggressive disease that responds poorly to conventional chemotherapy. Despite improvements in survival with the addition of tyrosine kinase inhibitors (TKI) to chemotherapy, hematopoietic allogeneic stem cell transplantation (ASCT) remains the only curative option in those eligible patients [1]. Molecular monitoring of *BCR-ABL1* transcripts is a valuable tool in assessing individual patient response to chemotherapy and ASCT [2–4]. The most common *BCR-ABL1* transcripts in Ph+ ALL are the e1a2, e13a2, and e14a2 fusions [5]; however, approximately 5% of adult patients express variant *BCR-ABL1* transcripts [6]. Of these variants, the e13a3 (b2a3) *BCR-ABL1* type is extremely rare with scant information regarding optimal therapeutic approach [7, 8]. Characterisation of these rare *BCR-ABL1* variants also affords the selection of appropriate primer/probe combinations for reverse-transcriptase quantitative PCR (RT-qPCR) assessment of residual disease. The presentation and clinical course of a patient with e13a3 *BCR-ABL1* Ph+ ALL is reported.

## 2. Case Report

**Figure 1:**
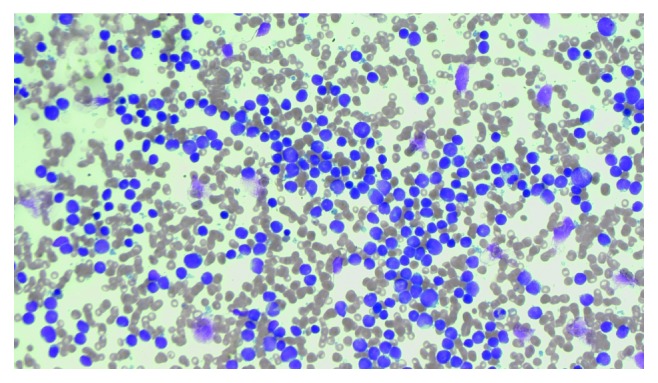
Bone marrow morphology at diagnosis showing infiltration by lymphoblasts.

**Figure 2:**
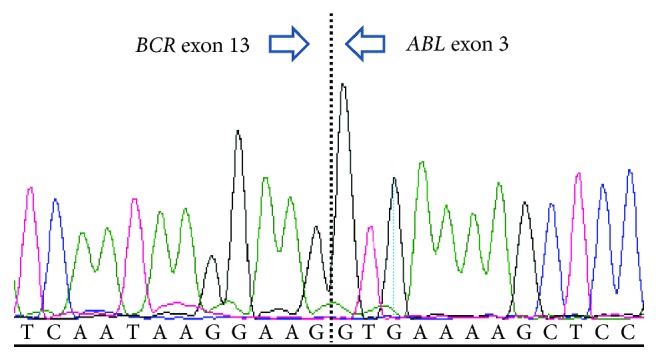
Sanger sequencing demonstrating presence of the e13a3 *BCR-ABL1* fusion gene.

A 23-year-old male presented with chest pain and dyspnoea, a hemoglobin count of 7.4 g/dL, platelet count of <10 × 10^9^/L, and white cell count of 11.6 × 10^9^/L. Bone marrow (BM) biopsy and aspirate demonstrated a 95% infiltration of lymphoblasts (Figure 1). Immunophenotyping of the BM aspirate showed lymphoblasts were CD10-, CD19-, CD20-, CD34-, TdT-, and HLA-DR-positive. Cytogenetic analysis revealed a complex clone in 10 cells analysed by G-banding containing various structural and numerical abnormalities including a derivative chromosome 22 from a translocation between the long arms of chromosomes 9 and 22. The composite karyotype was 44–48, *XY*, +*X*, *t*(1; 14)(p32; q32), −3, −6, add(6)(p21), add(7)(p21), add(8)(p21), −10, add(12)(p13), +16, add(19)(p13), der(22)*t*(9; 22)(q34; q11.2), +1∼2mar, inc [cp10]. Interphase FISH analysis showed the presence of *BCR-ABL1* rearrangement in 64/100 cells analysed. Standardised RT-PCR and Sanger sequencing demonstrated e13a3 *BCR-ABL1* transcripts [9] (Figure 2). E13a3 *BCR-ABL1* transcripts lack *ABL1* exon a2, thus prohibiting the use of a standardised primer/probe combination for e13a2/e14a2 *BCR-ABL1* qPCR [10]. A modified *BCR-ABL1* qPCR assay was therefore adopted utilising *BCR* forward primer ENF501F2 [9] with *ABL1* reverse primer ENR1063 and *ABL1* probe ENP1043, the latter both complementary to *ABL1* exon a3 sequence [11]. The *BCR-ABL1* standard curve was constructed using serial dilutions of an e14a3 *BCR-ABL1* plasmid over a five log range (100% to 0.001% *BCR-ABL1* transcripts) with *ABL1* as the reference gene as previously described [12]. Best practice guidelines for *BCR-ABL1* qPCR and data interpretation were followed [13]. Presentation e13a3 *BCR-ABL1* transcripts were of a high level (*BCR-ABL1*/*ABL1* 75.7%) consistent with a diagnosis of pre-pre-B cell Ph+ ALL.

**Figure 3:**
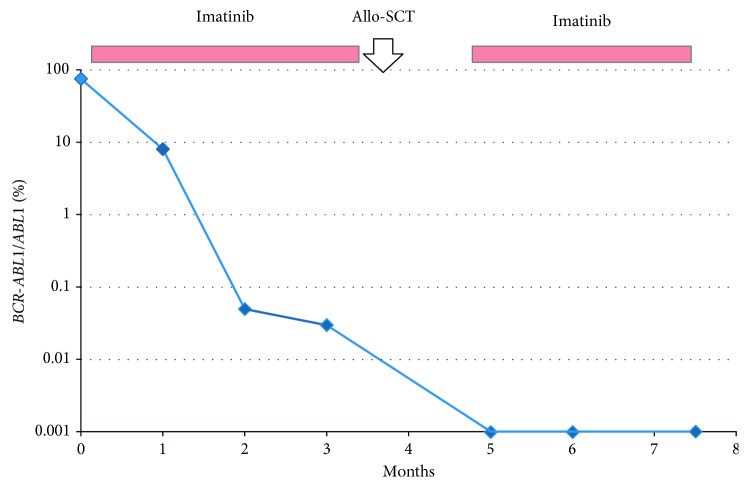
Molecular monitoring of e13a3 *BCR-ABL1* transcripts throughout clinical course. AlloSCT: allogeneic stem cell transplantation.

The patient commenced induction treatment with rituximab, dexamethasone, vincristine, and daunorubicin, with imatinib 400 mg oral daily started on day 15. After phase-one induction, the bone marrow aspirate demonstrated a complete morphological response with residual disease detected by immunophenotyping and RT-qPCR (*BCR-ABL1/ABL1* 8.11%). After phase-two induction, the bone marrow *BCR-ABL1/ABL1* level had fallen to 0.053%. Following high-dose methotrexate, the pre-ASCT *BCR-ABL1* level was 0.034%. The patient proceeded to ASCT from an unrelated donor after cyclophosphamide and total body irradiation conditioning and recommenced continued imatinib maintenance. *BCR-ABL1* transcripts were not detected in the peripheral blood at one, two, and three and a half months post-ASCT (Figure 3). Continued close molecular monitoring is planned.

## 3. Discussion

E13a3 *BCR-ABL1* transcripts lack *ABL1* exon a2 that encodes part of SH3 domain thought to contribute to leukemogenesis by inhibition of the kinase domain and by STAT5 activation [6]. In chronic myeloid leukemia patients, this transcript results in an indolent and TKI-responsive form of disease [14–16]; however, its prognostic significance in adult Ph+ ALL patients remains unknown due to the limited number of annotated cases. While the possibility exists of lymphoid blast crisis in chronic myeloid leukemia (CML), this transformation in e13a3 *BCR-ABL1* CML is rare [17]. In the absence of basophilia, thrombocytosis, and splenomegaly, this case likely represents *de novo* Ph+ ALL. Additional chromosomal abnormalities and complex karyotypes are frequently observed in Ph+ ALL, as witnessed in this case. There is some suggestion that in Ph+ ALL, additional cytogenetic abnormalities are associated with a shorter overall survival and might therefore be used for stratification purposes [18]. In the post-ASCT setting, monitoring *BCR-ABL1* transcript levels is an essential component of Ph+ ALL patient management [19] with effective standardisation of RT-qPCR assays for this and other variant *BCR-ABL1* fusion transcripts required [20].

Reporting of further cases would enable identification of any phenotypic characteristics of e13a3 *BCR-ABL1* Ph+ ALL and help establishing optimal treatment strategies for patients with this rare genotype.

## References

[1] Yilmaz M., Kantarjian H., Ravandi-Kashani F., Short N. J., Jabbour E. (2018). Philadelphia chromosome-positive acute lymphoblastic leukemia in adults: current treatments and future perspectives. *Clinical Advances in Hematology & Oncology*.

[2] Yanada M., Sugiura I., Takeuchi J. (2008). Prospective monitoring of BCR-ABL1 transcript levels in patients with Philadelphia chromosome-positive acute lymphoblastic leukaemia undergoing imatinib-combined chemotherapy. *British Journal of Haematology*.

[3] Zhang L., Ramjit R. T., Hill C. E., Arellano M., Khoury H. J., Mann K. P. (2016). Clinical significance of quantitative monitoring and mutational analysis of BCR-ABL1 transcript in Philadelphia chromosome positive B lymphoblastic leukemia. *Leukemia & Lymphoma*.

[4] Cazzaniga G., De Lorenzo P., Alten J. (2018). Predictive value of minimal residual disease in Philadelphia-chromosome-positive acute lymphoblastic leukemia treated with imatinib in the European intergroup study of post-induction treatment of Philadelphia-chromosome-positive acute lymphoblastic leukemia, based on immunoglobulin/T-cell receptor and BCR/ABL1 methodologies. *Haematologica*.

[5] Langabeer S. E. (2017). Variant BCR-ABL1 fusion genes in adult Philadelphia chromosome-positive B-cell acute lymphoblastic leukemia. *EXCLI Journal*.

[6] Tuszynski A., Dhut S., Young B. D. (1993). Detection and significance of bcr-abl mRNA transcripts and fusion proteins in Philadelphia-positive adult acute lymphoblastic leukemia. *Leukemia*.

[7] Burmeister T., Schwartz S., Taubald A. (2007). Atypical BCR-ABL mRNA transcripts in adult acute lymphoblastic leukemia. *Haematologica*.

[8] Zhang X., Pan J. (2016). An e13a3 BCR-ABL1 fusion transcript in variant t(9;22;17)(q34;q11;q21)-positive adult acute lymphoblastic leukemia. *International Journal of Laboratory Hematology*.

[9] van Dongen J. J. M, Macintyre E. A., Gabert J. A. (1999). Standardized RT-PCR analysis of fusion gene transcripts from chromosome aberrations in acute leukemia for detection of minimal residual disease. *Leukemia*.

[10] Gabert J., Beillard E., van der Velden V. H. J. (2003). Standardization and quality control studies of ‘real-time’ quantitative reverse transcriptase polymerase chain reaction of fusion gene transcripts for residual disease detection in leukemia—a Europe against cancer program. *Leukemia*.

[11] Beillard E., Pallisgaard N., van der Velden V. H. J. (2003). Evaluation of candidate control genes for diagnosis and residual disease detection in leukemic patients using ‘real-time’ quantitative reverse-transcriptase polymerase chain reaction (RQ-PCR)—a Europe against cancer program. *Leukemia*.

[12] McCarron S. L., Langabeer S. E., Bolger K. (2015). Molecular response to imatinib in chronic myeloid leukemia with a variant e13a3 BCR–ABL1 fusion. *Medical Oncology*.

[13] Foroni L., Wilson G., Gerrard G. (2011). Guidelines for the measurement of BCR-ABL1 transcripts in chronic myeloid leukaemia. *British Journal of Haematology*.

[14] Snyder D. S., McMahon R., Cohen S. R., Slovak M. L. (2004). Chronic myeloid leukemia with an e13a3 BCR-ABL fusion: benign course responsive to imatinib with an RT-PCR advisory. *American Journal of Hematology*.

[15] Pienkowska-Grela B., Woroniecka R., Solarska I. (2007). Complete cytogenetic and molecular response after imatinib treatment for chronic myeloid leukemia in a patient with atypical karyotype and BCR-ABL b2a3 transcript. *Cancer Genetics and Cytogenetics*.

[16] Liu B., Zhang W., Ma H. (2016). Complete cytogenetic response to Nilotinib in a chronic myeloid leukemia case with a rare e13a3(b2a3) BCR-ABL fusion transcript: a case report. *Molecular Medicine Reports*.

[17] Ha J., Cheong J.-W., Shin S., Lee S.-T., Choi J. R. (2016). Chronic myeloid leukemia with rare variant b2a3 (e13a3) BCR-ABL1 fusion. *Annals of Laboratory Medicine*.

[18] Seol C. A., Cho Y.-U., Jang S. (2017). Prognostic significance of recurrent additional chromosomal abnormalities in adult patients with Philadelphia chromosome-positive acute lymphoblastic leukemia. *Cancer Genetics*.

[19] Pfeifer H., Wassmann B., Bethge W. (2013). Randomized comparison of prophylactic and minimal residual disease-triggered imatinib after allogeneic stem cell transplantation for BCR-ABL1-positive acute lymphoblastic leukemia. *Leukemia*.

[20] Langabeer S. E. (2015). Standardized molecular monitoring for VariantBCR-ABL1Transcripts in chronic myeloid leukemia. *Archives of Pathology & Laboratory Medicine*.

